# Multi-scale Adaptive Computational Ghost Imaging

**DOI:** 10.1038/srep37013

**Published:** 2016-11-14

**Authors:** Shuai Sun, Wei-Tao Liu, Hui-Zu Lin, Er-Feng Zhang, Ji-Ying Liu, Quan Li, Ping-Xing Chen

**Affiliations:** 1 College of Science, National University of Defense Technology, Changsha, 410073, P. R. China; 2 Interdisciplinary Center of Quantum Information, National University of Defense Technology, Changsha, 410073, P. R. China

## Abstract

In some cases of imaging, wide spatial range and high spatial resolution are both required, which requests high performance of detection devices and huge resource consumption for data processing. We propose and demonstrate a multi-scale adaptive imaging method based on the idea of computational ghost imaging, which can obtain a rough outline of the whole scene with a wide range then accordingly find out the interested parts and achieve high-resolution details of those parts, by controlling the field of view and the transverse coherence width of the pseudo-thermal field illuminated on the scene with a spatial light modulator. Compared to typical ghost imaging, the resource consumption can be dramatically reduced using our scheme.

For imaging of a sparse scene, the request of both wide spatial range and high spatial resolution makes the resource consumption be huge for data acquiring, storage, delivery and processing. As a striking contrast, only few parts of the image are really interested, since in most cases we do not need all the details of the whole scene. If we can find out the location of interested objects before obtaining all the data, then achieve image of those parts with high resolution, it will be much more effective. Based on the development of computational ghost imaging (CGI)^1^,^2^, we propose and demonstrate a multi-scale adaptive imaging strategy to achieve this task.

Ghost imaging has been attracting more and more attention in recent years, which allows to reconstruct spatially resolved image with results of a single-pixel detector. In ghost imaging, two spatially correlated beams are employed. One beam illuminates the object, with the reflected (or transmitted) light collected by a bucket (single-pixel) detector. While the other beam, called reference beam, propagates directly to a spatially resolving detector, such as a CCD camera, with the intensity distribution recorded. By calculating correlation between results of two detectors, image of the object can be retrieved, while either detector can not provide the image by itself. Ghost imaging was firstly realized with entangled photons^3^,^4^, then demonstrated with pseudo-thermal source and true thermal source^5^,^6^,^7^,^8^,^9^,^10^,^11^,^12^,^13^,^14^,^15^,^16^. Since the function of the pixelated detector is to record the spatial distribution of the illumination pattern at each instant, the reference beam becomes obsolete if we can actively control the illumination pattern at the object position, with a device such as spatial light modulator (SLM)^1^,^2^. This is the main idea of CGI, which also offers idea of single-pixel camera. Based on idea of GI or CGI, 3-dimensional images have been reconstructed from single-pixel detectors^17^,^18^, information of a complex-value object can be obtained without lens^19^, and multi-spectral imaging methods using a single detector^20^ have been also developed. Besides, optical encryption were discussed as possible application of ghost imaging^21^.

Compared to GI, the size of data from the detector for CGI can be dramatically reduced, since there is only one pixel on the detector. However, many different illumination patterns are required for imaging and to be stored, with each frame being of the same size as the final image. The number of required patterns can be several thousands or even as high as one million. Even though compressed sensing (CS) offers a method to reduce the required number of frames^22^,^23^,^24^, it takes longer to reconstruct the image. To reduce the resource consumption, people tried in different ways, such as developing higher efficiency algorithms^24^,^25^,^26^,^27^,^28^,^29^, designing distributions of illumination patterns^17^,^30^,^31^,^32^, ameliorating the imaging tactics^33^,^34^,^35^ and so on.

Since an outline of the whole scene and high-resolution details of interested parts can be enough, we propose to achieve the image as follows. Firstly, obtain an image of the whole scene with low spatial resolution. Then, find out the interested parts and illuminate only those parts, for details of higher resolution. Adjust the range and the spatial resolution of illumination patterns according to the results, until the spatial resolution of the obtained image is high enough or reaches the limit of the imaging system. This is a multi-scale adaptive imaging strategy, which can be achieved based on the idea of CGI, with the resource consumption dramatically reduced.

## Results

### The multi-scale adaptive imaging scheme

**Figure 1:**
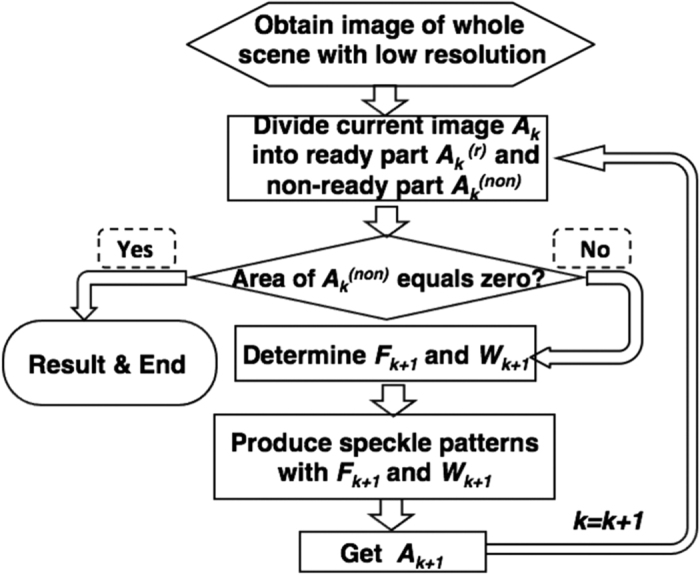
Flow diagram of our adaptive computational ghost imaging scheme. Each obtained image is divided into ready part and non-ready part. When the area of non-ready part equals zero, current image is the final output and the procedures end.

The outline of the whole scene and details of the interested parts are sequentially achieved with CGI, by adaptively controlling the field of view (FOV) and transverse coherence width (TCW) of the illumination patterns. A flow diagram of our adaptive imaging scheme is shown in Fig. 1.

1. To start, a ghost image *A*_*k*_(*k* = 1) of the whole scene with low spatial resolution is obtained.
2. From current image 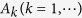, the interested parts are located and the whole image is divided into ready part 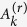 and non-ready part 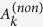.
3. If the area of 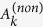 equals zero, the whole imaging process ends. Otherwise, go to next step.
4. From the parameters of the non-ready part, required FOV *F*_*k*+1_ and TCW *W*_*k*+1_ are determined.
5. Generate random patterns with settings of *F*_*k*+1_ and *W*_*k*+1_, and get image *A*_*k*+1_ using CGI. Then let *k* = *k* + 1 and go to step (2).

To demonstrate this scheme, we have to control the FOV and TCW of the speckle patterns. FOV defines the range of current imaging, and TCW is equivalent to the average size of speckles. Experimentally, FOV and TCW of speckle patterns can be tailored by a SLM controlling the phase at every pixel lighted by the wavefront of the laser beam. To obtain the phase masks used to generate speckle patterns of required FOV and TCW, random speckle patterns of required parameters are numerically generated and serve as objective functions, from which the phase masks can be numerically obtained with the GS algorithm^36^. What’s more, we have to determine every part of current image is ready or not, which can be done by locating the interested part and finding out whether the resolution of interested parts is appropriate for the target. For each image obtained in step (5), we check the normalized value of the image against a threshold *h*_1_, which is selected according to Gray-Level Histograms Method^37^. The region where the value of image is smaller than *h*_1_ is taken as ready part. At the same time, we compare correspondence part of images *A*_*k*_ and *A*_*k*−1_ (*k* > 1) to determine whether the spatial resolution is high enough for every interested part. If Mean Square Error (MSE) between them is smaller than an empirical threshold *s*, the resolution of *A*_*k*_ is high enough, thus this part will be also taken as ready (see Method section for details).

For a given scene, the Contrast-to-Noise Ratio (CNR) of the ghost image is proportional to 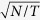, with *N* being the number of illumination patterns (or number of measurements) and *T* being the mean number of speckles that light into the area of the object^38^. Therefore, to obtain an image of certain CNR, the number of required patterns can be reduced if the mean number of speckles can be reduced. Or, image of lower spatial resolution requires smaller number of illumination patterns. Besides, finding out the interested parts and illuminating only those parts can reduce the range of illumination patterns. Then the size of measurement matrix for reconstruction of image can also be reduced, since those matrix are actually numeric expression of those illumination patterns. Therefore the resource consumption of data processing and storage can be reduced greatly. In multi-scale adaptive ghost imaging, FOV and TCW of the speckle patterns are adaptively adjusted according to acquired information from previous measurements, rather than using the speckle patterns with smallest TCW that can be generated by the optical system all the way, so an image with higher CNR and high enough resolution can be reconstructed from fewer number of patterns. In our adaptive imaging strategy, speckle patterns of low spatial resolution are employed to reduce the number of required patterns for the whole range, while speckle patterns of high spatial resolution and small FOV are employed to get details and reduce the size of measurement matrices at the same time. Therefore, compared with traditional GI, multi-scale adaptive ghost imaging can help to reduce resource consumption greatly.

### Experimental results

**Figure 2:**
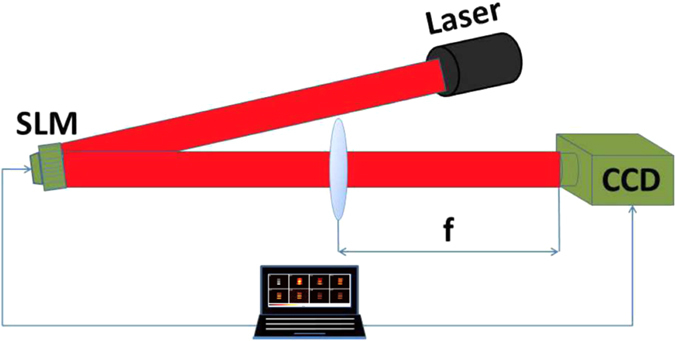
Illustration of the experimental setup. A laser beam illuminates an SLM working in phase-only mode, which contains 512 × 512 addressable pixels. The lens (*f* = 20 *cm*) in the optical system serves as a Fourier transformer with a CCD camera placed at the back focal plane of the lens. This CCD serves for the considered scene and as the bucket detector.

We build an experimental setup, with a diagram shown in Fig. 2. A laser beam with wavelength of *λ* = 1064 *nm* illuminates an SLM (BNS P512-1064) working in phase-only mode. The beam is treated as plane-wave, which is expanded by a beam expander to illuminate all the 512 × 512 addressable 15 × 15 *μm*^2^ pixels of the SLM. There is a lens (*f* = 20 *cm*) following the SLM, serving for optical Fourier transformation. A CCD camera is placed at the back focal plane of the lens, which consists of 400 × 400 addressable 5.5 × 5.5 *μm*^2^ pixels. The speckle patterns generated by the SLM are recorded by the CCD. The sparse scene to be imaged is simulated with a virtual binary picture with a size of 400 × 400. Pixel-to-pixel multiplication is done between the virtual picture and the intensity of speckle patterns recorded by the CCD, with all the products accumulated to serve as the bucket signal. The corresponding reference speckle patterns are calculated from the phase masks imprinted on the SLM. Ghost image of the virtual scene is reconstructed by calculating the correlation between the reference patterns and the bucket signal. Resulted from the finite filling fraction of the pixels of SLM, there exists a cross hot spot in the generated field. In order to separate the speckle patterns from the cross hot spot, each modulated phase pixel is realized by a combination of 4 × 4 physical pixels on the SLM. Therefore, a phase mask with a size of 128 × 128 is loaded to the SLM for each measurement in our experiment. The resolution limitation of our system is *R*_*p*_ = *λf*/*D*, where *D* is the clear aperture of SLM, which is 7.68 mm.

**Figure 3:**
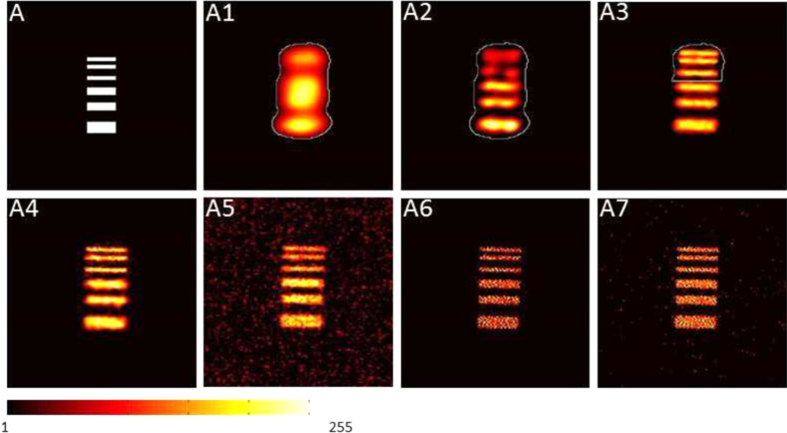
Results of adaptive computational ghost imaging. A is the scene to be detected. A1–A4 are sequential results retrieved from intensity correlation of 1000, 2000, 4000, 8000 frames of speckle patterns, with the corresponding TCW of the illumination field set as 8*R*_*p*_, 4*R*_*p*_, 2*R*_*p*_ and *R*_*p*_, respectively. A4 is also the final result. The closed solid curve in A1–A3 encloses the non-ready part, as well as the field of view for next step. A5 is the image obtained from typical ghost imaging using 15000 frames of speckles without feedback strategy, with TCW of the illumination field set to be *R*_*p*_. A6 is retrieved from CS algorithm using 9000 frames of speckle patterns, with transverse coherence width being *R*_*p*_ and FOV being the non-ready part shown in A1. As a comparison, A7 is reconstructed from 10000 frames of speckles with transverse coherence width of *R*_*p*_ and FOV being the area of the whole scene.

To investigate the performance of our adaptive imaging method, ghost images of a virtual scene (shown in Fig. 3A) are reconstructed with intensity correlation algorithm, with the results shown in Fig. 3. *Ak*(*k* = 1, 2, 3, 4) are sequential results retrieved using speckle patterns with TCW of 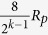, and the number of used speckle patterns are 1000, 2000, 4000 and 8000, respectively. From A1 to A4, FOV of the illumination field is also adaptively controlled. The non-ready part of current image, which is also the illumination area for next step, is enclosed with the closed solid curve in A1-A3. To determine the area of non-ready part, the normalized value of image are compared to a threshold according to Gray-Level Histograms method, and MSE between current image and the previous one is compared to an empirical threshold. A5 is a ghost image of the whole scene reconstructed using 15000 speckle patterns with transverse coherence width of *R*_*p*_. By comparing A4 and A5, it shows that the background noise can be effectively removed with adaptive ghost imaging method, since FOV of most speckle patterns are limited around the object. To be more precise, we calculated CNR of A4 and A5 within the region where A2 is illuminated, with the results being 4.82 for A5 and 6.88 for A4. That is, our adaptive ghost imaging method can work well and provides image of higher quality.

**Figure 4:**
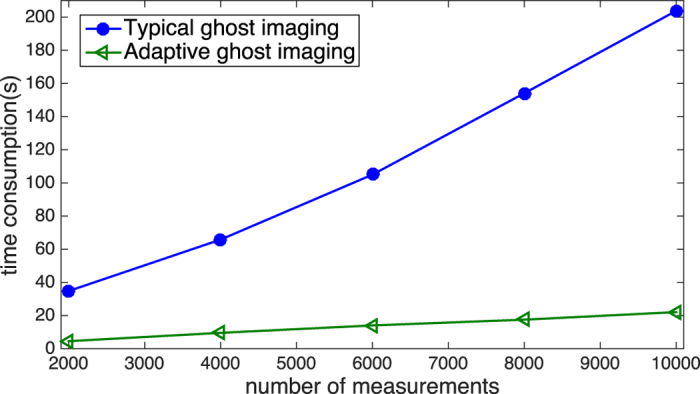
The time consumption of CS algorithm with varying number of measurements. The dots show time consumption of CS algorithm to reconstruct image of A without feedback strategy. The triangles show time consumption of CS algorithm in adaptive CGI scheme.

The performance of adaptive ghost imaging with CS algorithm is also investigated. During each run, the speckle patterns are randomly generated for given TCW and FOV. That is, the spatial distribution of the intensity within the area of FOV is random, which satisfies the restricted isometry property condition. For speckle patterns used in different runs, since they are illuminating at different area (different FOV) with different resolution (different TCW), CS algorithm can also work although those patterns appear somehow in sequence according to the spatial resolution. Illuminate the object with low-resolution speckles, we can obtain the outline of the interested part from a small number of measurements, which helps to determine FOV of high-resolution speckle field. With FOV of high-resolution patterns limited to the interested region, the data size of the measurement matrices and the time consumption for the convex optimization is greatly reduced. Based on gradient projection for sparse reconstruction^39^ in two dimensional discrete cosine transform (2D-DCT) domain, images from our method and traditional ghost imaging are reconstructed, with an example shown in Fig. 3. A6 shows the result of adaptive imaging, reconstructed from 10000 speckle patterns of different settings, 1000 of which illuminate on the whole scene with resolution of 8*R*_*p*_ and 9000 of them illuminate only on the non-ready part shown in A1 with resolution of *R*_*p*_. CS algorithm is only used for those patterns of smaller FOV, to enhance the quality of image. A7 is the image reconstructed via CS with 10000 speckle patterns of the same setting, illuminating the whole scene with resolution of *R*_*p*_. Therefore, the spatial resolution of A6 and A7 are the same. For A7, the data size of measurement matrix is 13.14 GB and it takes 203.8 seconds to get the final result on our PC, which consists of an Intel Xeon CPU @ 2.10 GHz and a 96 GB RAM. While for A6, the data size of the measurement matrices is 2.86 GB and it takes 20.7 seconds to get the output. That is, the data size (or the consumption of storage) and time consumption for CS reconstruction are much smaller using adaptive scheme. Further more, we record the time consumption of CS algorithm for different numbers of measurements, also for the scene of picture A. The results are shown in Fig. 4. Those triangles show time consumption for adaptive CGI case and those dots show time consumption for GI without feedback strategy, respectively. These results also demonstrate that adaptive CGI can greatly reduce the time consumption, and the time consumption increase much slower with ascending number of measurements.

## Discussion

Multi-scale adaptive ghost imaging offered a seminal idea that data analysis can be conducted before the data acquisition is completed. That is, one can analyze data as soon as possible in the imaging process, pay close attention to the feedback information of the interested area and take it as a prior information to adaptively control the detecting system, which can make the data acquisition devices coordinate with the data analyzing system, resulting in resource saving and more effective information acquisition. In our experiments, intensity on each pixel of the obtained image in each step is used to determine the ready and non-ready part. If the object has a continuously varying gray scale, systematic errors will be introduced for those parts where grey value is very close to that of background due to our settings of threshold. Further more, if the scene is not spatially sparse, our method can not be effective in reducing resource consumption. However, we believe the idea of adaptive imaging can still work, so long as the acquired data provide meaningful information about the object and the scene is sparse in a specific space. Other information of the object, such as velocity, can also be useful for design of next illumination patterns. At the same time, optimization of the adaptive strategy might reduce the resource consumption more. Here we only show an instance, more possibilities of our method are quite open.

In addition, to implement multi-scale ghost imaging, it is also possible to place the modulating device behind the object. Alternately, the object is uniformly illuminated with the transmitted light being structured by an SLM or other modulation devices. Performance and possible applications of the imaging system will be somewhat different, using two different configurations. With transmitted light structured, less and less part of the light energy from the object is useful, when FOV is setting smaller and smaller. Further more, the spatial resolution will be determined by the clear aperture of lens and resolution of the modulator. While, with illumination field structured, all the energy from the source are lighted onto the interested area by controlling FOV, and the spatial resolution is determined by the lighted aperture of the modulator and aperture of the lens. However, it can not be used for passive imaging.

In conclusion, a multi-scale adaptive computational ghost imaging scheme is proposed and demonstrated. By controlling the phase mask loaded on an SLM, TCW and FOV of the generated pseudo-thermal source vary according to previous imaging results. Speckle patterns of large TCW were produced to get the outline of whole scene, from which the interested area can be determined. Then FOV of the illumination field was limited around the interested part and speckle patterns of smaller TCW were used to reconstruct details of interested area. TCW and FOV of the illumination patterns are adaptively controlled during all the sequential procedures. Compared with traditional ghost imaging, resource consumption of multi-scale adaptive ghost imaging can be dramatically reduced since we only get details of the interested parts. This method will help to reconstruct the ghost image of interested area quickly in a sparse scene. It may help to push the application of ghost imaging in the cases of Earth observation, searching over a wide range, imaging into the sky and so on.

## Methods

To determine whether each part of the obtained image is ready or not, MSE between current image and the previous one within the considered part is calculated. If the MSE is small enough, the current image of the considered part is taken as ready. This is effective and practical, based on an observation that MSE between an obtained ghost image and the real scene reaches its minimum when the average size of speckles is close to feature size of the object, reconstructed from a given number of speckle patterns.

**Figure 5:**
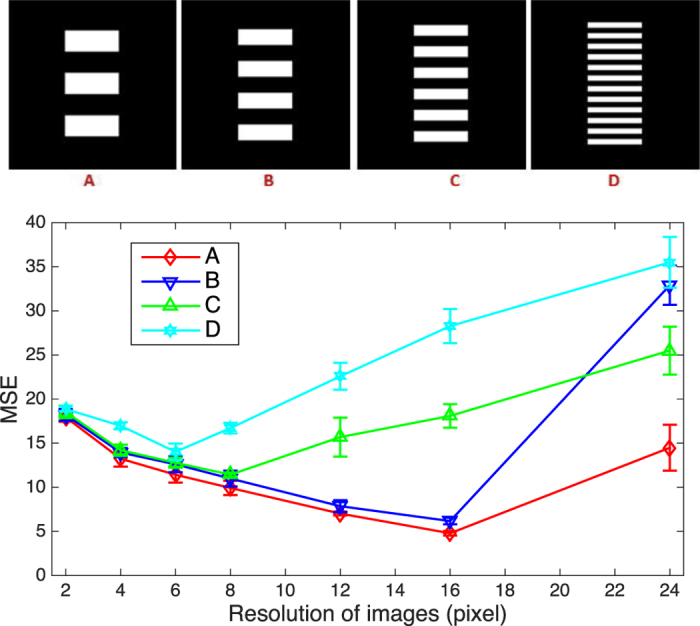
Under given number of measurements, the behavior of MSE with varying resolution of imaging is investigated, employing objects (**A**–**D**). For each object, MSE reaches its minimum when the resolution of image is close to feature size of the object.

MSE is a parameter to evaluate the quality of the image, which follows the expression 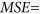 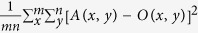, where *A*(*x, y*) is the ghost image and *O*(*x, y*) is the reflection (or transmission) rate distribution of the scene, with *m* and *n* being the indices of the pixels. The higher the quality of image, the smaller is MSE, and the image is closer to the real scene. Since ghost imaging can also be taken as a method of statistical imaging, the image quality is related to the number of used speckle patterns and the resolution of the illumination field. For illumination field of given TCW, MSE will decrease with increasing number of used speckle patterns. However, for given number of speckle patterns, MSE is not monotonic. If the size of speckles is larger than feature size of the object, the obtained image contains more information about details of the object if using smaller speckles and MSE will descend if the size of speckles decreases. While the size of speckles is smaller than feature size of the object, noise of high spatial frequency will be more difficult to be suppressed with given number of measurements, therefore MSE will increase with decreasing TCW. Based on this observation, MSE between *k*^*th*^ and (*k* + 1)^*th*^ normalized ghost image 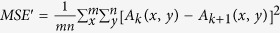, also reaches its minimum when TCW of the illumination field is close to feature size of the object. We did numerical simulation to verify this. Four sets of binary stripes of different feature sizes are considered, as shown in Fig. 5, with the center to center distance between adjacent stripes set to be 32 pixels, 24 pixels, 16 pixels and 8 pixels, respectively. For each object, random speckle patterns of different TCW, *R*_*k*_ ∈ [1, 2, 4, 6, 8, 12, 16, 24] pixels, are generated for ghost imaging and the number of illumination patterns are set to ensure that the obtained images with different resolution have an approximately equivalent CNR. MSE_*k*_ between images with resolution of *R*_*k*_ and *R*_*k*−1_ are calculated and shown in Fig. 5. The results show that MSE reaches its minimum when the resolution of the illumination filed is close to feature size of the object.

Based on this, in step (2), MSE between current image and previous image is calculated for each part to determine whether this part is ready or not. If it is smaller than an empirical threshold, the resolution of this part is taken as high enough, thus this part will be set as ready.

## Additional Information

**How to cite this article**: Sun, S. *et al*. Multi-scale Adaptive Computational Ghost Imaging. *Sci. Rep.* **6**, 37013; doi: 10.1038/srep37013 (2016).

**Publisher’s note:** Springer Nature remains neutral with regard to jurisdictional claims in published maps and institutional affiliations.
